# Supplementary material for: Standing Meetings Are Feasible and Effective in Reducing Sitting Time among Office Workers—Walking Meetings Are Not: Mixed-Methods Results on the Feasibility and Effectiveness of Active Meetings Based on Data from the “Take a Stand!” Study
Source: Int J Environ Res Public Health. 2020 Mar 5;17(5):1713. doi: 10.3390/ijerph17051713 (PMC7084221; doi:10.3390/ijerph17051713)
Supplement: Supplementary file 1 [file ijerph-17-01713-s001.pdf]

Supplementary material

	N (%)	Sitting at 3 mo[a] (CI 95%)
**Participation in standing meetings**		
No	9 (7)	
Yes, occasionally	66 (52)	-23 (-64;17)
Yes, regularly	52 (41)	**-59 (-102;-17)**
**Participation in walking meetings**		
No	69 (55)	
Yes, occasionally	55 (44)	-15 (-38;7)
Yes, regularly	2 (2)	-55 (-141,30)

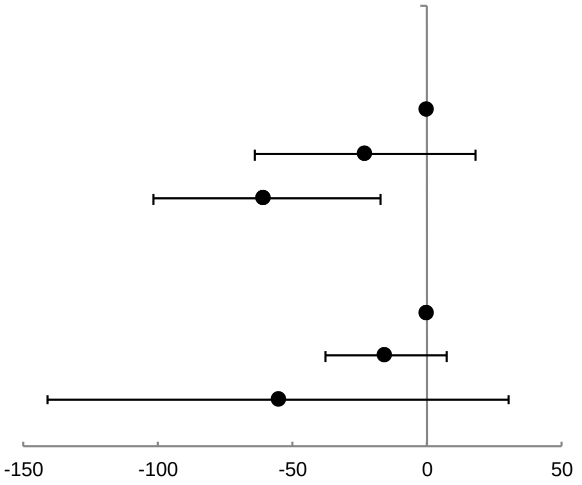

Figure S1. Sensitivity analysis: Model controlled for leisure time physical activity. Participation in standing and walking meetings and association with sitting time at 3 months follow-up (n = 127). Boldface indicates statistical significance (p = 0.006). [a] Sitting/8 h workday at 3 months follow-up compared to baseline. The model included participation in standing and walking meetings as the exposure; included sitting time at 3 months as the outcome; and controlled for sex, age, baseline sitting time and objectively measured total physical activity at baseline.

	N (%)	Sitting at 3 mo[a] (CI 95%)
**Participation in standing meetings**		
No	9 (7)	
Yes, occasionally	66 (52)	-24 (-65;16)
Yes, regularly	52 (41)	**-61 (-103;-19)**
**Participation in walking meetings**		
No	69 (55)	
Yes, occasionally	55 (44)	-16 (-38;6)
Yes, regularly	2 (2)	-54 (-140,31)

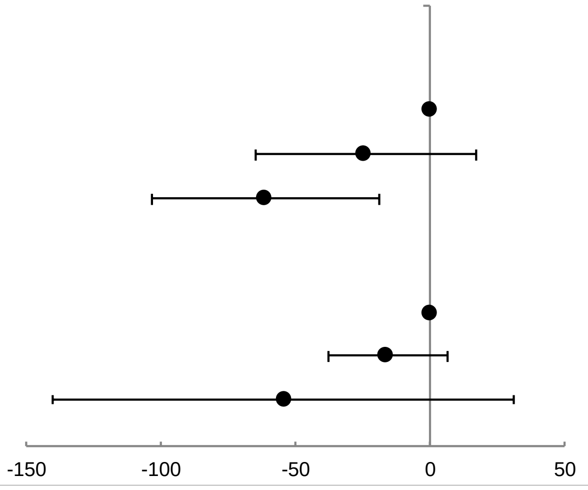

Figure S2. Sensitivity analysis: Model controlled for leisure time steps. Participation in standing and walking meetings and association with sitting time at 3 months follow-up (n = 127). Boldface indicates statistical significance (p = 0.005). [a] Sitting/8 h workday at 3 months follow-up compared to baseline. The model included participation in standing and walking meetings as the exposure; included sitting time at 3 months as the outcome; and controlled for sex, age, baseline sitting time and objectively measured leisure time steps at baseline.
